# Multi-Objective Optimal Allocation of Water Resources Based on the NSGA-2 Algorithm While Considering Intergenerational Equity: A Case Study of the Middle and Upper Reaches of Huaihe River Basin, China

**DOI:** 10.3390/ijerph17249289

**Published:** 2020-12-11

**Authors:** Jitao Zhang, Zengchuan Dong, Tian Chen

**Affiliations:** College of Hydrology and Water Resources, Hohai University, Nanjing 210098, China; jtzhang@hhu.edu.cn (J.Z.); chentian0318@hhu.edu.cn (T.C.)

**Keywords:** intergenerational equity, sustainable development, discount value, conflict resolution, multi-objective optimal allocation of water resources, Huaihe River Basin

## Abstract

With the rapid development of society and the economy, the demand for water resources is increasing. This, combined with the increasing competition for water resources between current and future generations, hinders the sustainable development of society. To alleviate prominent water resources problems, achieve sustainable utilization of water resources and the sustainable development of society and economy, a multi-objective optimal water resources allocation model is proposed, in which different water sources and different water departments are considered to achieve the maximum social and economic benefits of the study area on the premise of water resources sustainability. To meet the needs of future generations, the discount value is introduced to measure intergenerational equity. A case study from seven cities in the upper and middle reaches of the Huaihe River Basin is given to verify the practicality and viability of the model. The non-dominated sorting Genetic Algorithms-2(NSGA-2) was used to find optimal water resources allocation schemes in 2020 and 2050 under the condition of a hydrological drought year (inflow guarantee rate *p* = 75%). Compared with previous models, the intergenerational equity model considers the sustainability of water resources, has higher social and economic benefits, and ensures the fair distribution of water resources among generations. According to the results, under balanced weight, the water shortage ratio of the seven cities will decrease from 5.24% in 2050 to 1.58% in 2020, and the economic benefit will increase from 79.46(10^10^CNY) to 168.3(10^10^CNY), respectively. In addition, the discount value of economic benefit in 2050 is 80.23(10^10^CNY), which is still higher than that in 2020. This shows that the water resource allocation scheme can eliminate the disparity between supply and demand for water resources and achieve intergenerational equity. Therefore, the intergenerational equity model can alleviate the contradiction of water resources and realize intergenerational equity.

## 1. Introduction

Water is necessary for social and economic development. However, with the growth of population and the rapid development of social economy, the problems of water shortage and water pollution are becoming increasingly prominent [1]. This has aroused people’s concern about the shortage and unreasonable distribution of water resources [2]. Therefore, the optimal allocation of water resources, water resources carrying capacity and river ecological flow have become the hot issues [3,4].

The study of the optimal allocation of water resources began in the 1950s. In the past few decades, it has become the focus of more and more scholars, and the allocation of water resources in trans-boundary basins is also the focus of international attention [5]. Qiu et al. established flood resource allocation considering ecological benefits to achieve effective utilization of flood resources [6]. Li et al. established the optimal allocation model for water resources in irrigated areas and considered the uncertainty of water resources to solve the problem of agricultural water shortage [7]. Li et al. also implemented another water resources allocation model based on Nash negotiation. This model was applied to the Lancang–Mekong River basin to solve the water resources allocation problems in China, Thailand and Myanmar in 2020 [8]. Chen et al. study the relationship among the water resources system, energy and food, and take Ordos as the research area to carry out water resources allocation, providing suggestions for the sustainable development of the study area [9]. In the allocation of water resources, efficiency and equity are important factors. The efficiency of water resources allocation reflects the economic benefits of water resources allocation, whereas the equity of water resources allocation balances the relationship between supply and demand and reflects the social benefits brought by water resources [10]. Only scientific and well planned allocation of water resources to each water sector will produce economic and social benefits [11,12]. In terms of efficiency, Xu et al. use economic benefits as one of the objective functions to measure the efficiency of water resources allocation [13]. Jiang et al. constructed a simulation model of pond water allocation in the irrigation area to maximize the benefit of agricultural water use based on the water resources allocation [14]. In relation of equity, Alberto et al. solve the problem of allocation from the perspective of water rights trading [15]. Zhang et al. evaluate whether the water supply and demand match in space to judge whether the allocation is fair [16]. Additionally, Chen et al. introduce the water shortage rate as social benefits function to measure the fairness of allocation [17]. These ideas of water resources allocation have been proven to be reasonable and effective. However, these methods do not solve the problem from the perspective of intergenerational equity. The conflict of water use between contemporary and future generations is still an obstacle to the improvement of water resources allocation models.

At present, study on water resources allocation methods is becoming more comprehensive, but research on intergenerational equity is still in the development stage. When thinking of the future, allocation of water resources needs to include the concept of sustainable development, and sustainable development needs to consider the needs of current and future generations of water resources [18]. This is the concept of intergenerational equity. Intergenerational equity should be incorporated into management strategies as an important concept. In this way, people in the present can realize that unreasonable water resources allocation schemes will bring risks to future generations’ water use.

Some researchers have considered this problem. For example, Gleeson et al. take groundwater resources as value driven to promote intergenerational equity [19]; Xu et al. introduce the Gini coefficient to establish a model that considers intergenerational equity, and successfully test the model with the Minjiang River Basin [13]. Australia’s Murdering Basin has set up a special institution to carry out water supply resource management, specifically to address the problem of intergenerational equity [20]. Some scholars want to protect the river channel through engineering measures to achieve intergenerational equity. Bahrami-Yarahmadi et al. study the scour patterns around different spur dikes, trying to direct the flow to the middle of the river to reduce the erosion of the river banks, achieve the balance of ecological habitat protection, protect the river environment and realize sustainable development [21]. Although intergenerational equity is a key part of the international community’s commitment to the future, there is no uniform strategy to solve water competition between current and future generations.

Some indicators are used to characterize sustainability in economics, and discount value is one of them [22]. The discount value is used to measure intergenerational equity of non-renewable resources allocation. Discount value not only affects economic growth [23], but is also a common measure used to measure the value of resources in different periods and demonstrate the quality of nature resources’ intertemporal allocation [24]. Sieveking and Semmler [25] discuss the allocation results of resources at large discount rate and small discount rate, respectively. Farzin [26] posits that the discount rate has a very close relationship with the rate of resource consumption. Rodriguez et al. [27] evaluated the applicability of the discounted value model as an intertemporal selection process. Therefore, according to previous research, this paper introduces the discount value to water resources allocation, combined with water shortage rate and pollutant emissions, to measure the intergenerational equity of water resources allocation.

With the rapid development of computer technology, a large number of intelligent optimization algorithms are used to solve multi-objective optimization problems, such as the Particle Swarm Optimization algorithm (PSO) and the Genetic Algorithms (GA) [17]. In this paper, the non-dominated sorting Genetic Algorithms-2 (NSGA-2) is used to calculate the multi-objective optimal allocation model of water resources. The NSGA-2 algorithm originated from the GA algorithm, and was then improved by Deb to form the NSGA-2 algorithm [28]. The NSGA-2 is a global optimization probability algorithm. Compared with the traditional single point search algorithm, it can avoid falling into local optimum and has better global search performance. Moreover, the NSGA-2 algorithm has low requirements in terms of the form of objective function and constraint conditions [29,30]. Gu et al. use the NSGA-2 algorithm to solve the multi-objective optimization problem [31]. Therefore, this paper introduces the NSGA-2 algorithm to solve the multi-objective optimal allocation of water resources problem.

To alleviate the competition between water departments and solve the conflicts between generations, this paper establishes an optimal allocation model of water resources based on intergenerational equity, which considers efficiency, equity, and sustainable development. In order to meet the demands of rapid economic development, one of the objectives of the model is to maximize water use income. To meet the water demand of the water sector as fully as possible, the model takes the minimizing the water shortage rate (ratio of water shortage to water demand) as one of its objectives. In addition, this paper introduces the idea of discount value (the present value of the monetary value of future benefit), and considers the ecological benefits (at present, the quantification of ecological benefits is still in the development stage, so this paper takes pollutant emissions as the representative of ecological benefits), so as to ensure water resources regeneration, which will lead to intergenerational equity.

This paper will make the following contributions to the literature. First of all, this paper interprets the connotation of intergenerational equity in water resources allocation; this paper believes that intergenerational equity is not only the fairness of water resources quantity, but also the equity of water resources quality and efficiency. Secondly, this paper introduces the discount value and pollutant emissions into the water resources allocation model, which promotes the intergenerational equity of water resources allocation. Thirdly, this paper constructs an optimal allocation model of water resources including efficiency, fairness and sustainable development, which provides a reasonable strategy for basin water resources management. Finally, this paper uses the NSGA-2 algorithm to solve the multi-objective optimal allocation model of water resources and proposes water resources allocation schemes for the seven cities of the Huaihe River Basin.

## 2. Methodology and Materials

### 2.1. Key Problem Statement

**Figure 1:**
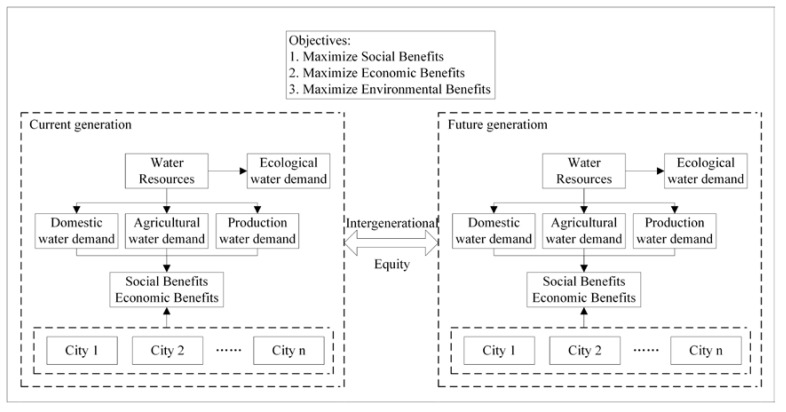
Flowchart of the methodology.

With the continuous growth of population, the environment is rapidly being destroyed, people have higher demand for water resources, and the international community pays more attention to intergenerational equity, which poses a new challenge to the existing water resources management strategy. To achieve sustainable development, protect the rights and interests of future generations of water resources and realize intergenerational equity, it is necessary to create a new water resources allocation model. One of the methods to solve the problem of water resource management is multi-objective optimization [32]. This paper constructs a water resource allocation model that includes efficiency, equity, and sustainable development, with a focus on promoting intergenerational equity. The flow chart of the proposed method is shown in Figure 1.

### 2.2. Scenario Setting

The area of study is the 7 cities of the middle and upper areas of the Huaihe River Basin. This paper uses the intergenerational equity-based water resources collaborative allocation model to study the allocation of water resources under the inflow guarantee rate of *p* = 75% in 2020 and 2050. In this paper, 2020 and 2050 are set as two intergenerational representative years, with different levels of social development. This paper sets the inflow guarantee rate of *p* = 75% in 2020 as scenario 1 and *p* = 75% in 2050 as scenario 2.

### 2.3. Area of Study

**Figure 2:**
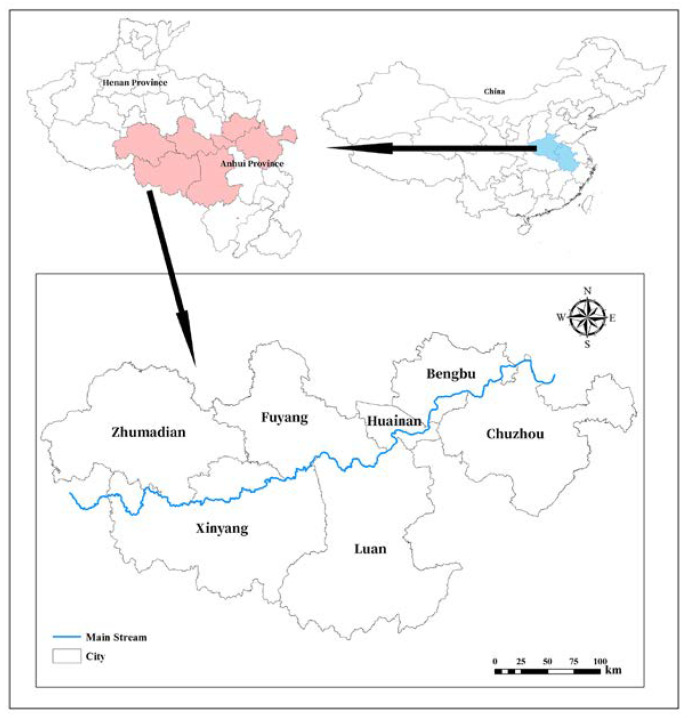
Location of the study area.

As shown in Figure 2, the Huaihe River Basin is located at 112°–121° E and 31°–36° N. It is located between the Yellow River Basin and the Yangtze River Basin, with a drainage area of 270,000 km^2^. It includes the four provinces of Henan, Anhui, Jiangsu, and Shandong, and is an important food and manufacturing base in China. However, the middle and upper reaches of the Huaihe River Basin have a large population and belong to the high population density area. The level of economic development and urbanization is less advanced in China. According to the water resources bulletin of the Huaihe River area, the average precipitation in the middle and upper reaches of the Huaihe River Basin is 700 mm. The precipitation time is mainly concentrated in June to September, and the space is mainly concentrated in the lower reaches of the Huaihe River Basin. The precipitation presents uneven spatial and temporal distribution. Due to the severe water shortage area, the per capita water resources are less than 500 m^3^. In recent years, the water resources in the upper and middle reaches of the Huaihe River Basin have been supplemented by the Yangtze River Basin [33,34,35].

However, with the continuous development of social and economy, the demand for water resources in the middle and upper reaches of Huaihe River Basin is getting higher and higher. This is intensifying the competition for water resources between different water use departments. Solving this problem requires the optimization of the allocation of water resources in the middle and upper reaches of Huaihe River Basin. This will alleviate the disparity between supply and demand of water resources and improve the social, economic and environmental benefits generated by water resources. This is of great practical significance for realizing intergenerational equity of water resources and sustainable development of the middle and upper reaches of the Huaihe River Basin.

The Huaihe River Basin is an important grain and industrial base in China. Therefore, the main water consumption departments in the middle and upper reaches of the Huaihe River basin can be divided into domestic water, agricultural water, production water and ecological water; the production water includes the water for the secondary industry and the tertiary industry. According to the planning of Huaihe River Basin Management Committee, in 2020, the benefit coefficient of domestic water, agricultural water and production water in the middle and upper reaches of Huaihe River Basin are 40 CNY/m^3^, 55.6 CNY/m^3^ and 78.9 CNY/m^3^, respectively; in 2050, the benefit coefficient of domestic water, agricultural water and production water will be 60 CNY/m^3^, 125 CNY/m^3^ and 153 CNY/m^3^, respectively. According to the bank’s benchmark interest rate, the discount rate is 2.5% (source: http://www.hrc.gov.cn/main/szygb/21448.jhtml:).

### 2.4. Data Source

In this study, water resource data and socio-economic data are sourced from the Huaihe River Basin Water Resources Bulletin (source: http://www.hrc.gov.cn/main/szygb/21448.jhtml), and the Statistical Yearbook published by the Anhui provincial government, Henan Provincial Government and China National Bureau of Statistics (source: http://tjj.ah.gov.cn/ssah/qwfbjd/tjnj/index.html, http://www.ha.stats.gov.cn/tjfw/tjcbw/tjnj/, http://www.stats.gov.cn/tjsj/ndsj/).

## 3. Modeling

In this paper, a multi-objective allocation model of water resources is established. The purpose is to make a suitable water resources allocation scheme for the middle and upper reaches of Huaihe River Basin, so as to realize the sustainable utilization of water resources and the sustainable development of society.

### 3.1. Model Construction

The model considers four water consumption sectors: domestic water, agricultural water, production water and ecological water. The water supply sources are summarized as local surface water, local groundwater, unconventional water, and water distributed from other basins.

#### 3.1.1. Objective Functions

(1) Maximize Social Benefits

Following the principle of equity and efficiency, in order to improve the social benefits of water distribution, and taking the minimum water shortage of the water supply system in the area of study as the goal:

(1) $$\min f_{1}(Q)={\sum_{j=1}^{J}\sum_{k=1}^{K}\left(\frac{D_{jk}-\sum_{t}^{T}\sum_{i=1}^{I}Q_{ijkt}}{D_{jk}}\right)}^{2}$$

where $D_{jk}$ denotes the water demand of the water consumption department k of the calculation unit j. $Q_{ijkt}$ is the water supply quantity of water source i to water consumption department k of calculation unit j in period t.

(2) Maximize Economic Benefits

Many researchers have studied economic benefits, and some have proposed the calculation of economic benefits from the demand function [10,36]. In this paper, the economic benefit and cost of per unit water volume are used to calculate economic benefit.

Following the principle of high efficiency, in order to improve the output value of the unit water supply, and taking the maximum economic benefit of the research area as the goal:

(2) $$\max f_{2}(Q)=\sum_{t=1}^{T}\sum_{i=1}^{I}\sum_{j=1}^{J}\sum_{k=1}^{K}\left(b_{ijk}Q_{ijkt}-c_{ijk}Q_{ijkt}\right)$$

In the formula, $b_{ijk}$ is the water supply benefit ($\mathrm{CNY}/m^{3}$) from the water source i to the water consumption department of the calculation unit j, and $c_{ijk}$ is the water supply cost ($\mathrm{CNY}/m^{3}$) from the water source i to the water consumption department of the calculation unit j.

(3) Maximize Environmental Benefits

Following the principle of sustainability, in order to reduce the discharge of waste water into the river and promote a healthy cycle of development for the environment, and taking the minimum sum of COD discharge of waste water into the river for each calculation unit as the goal:

(3) $$\min f_{3}(Q)=\sum_{j=1}^{J}\sum_{k=1}^{K}0.1\cdot d_{jk}p_{jk}\sum_{i=1}^{I}Q_{ijk}$$

where $d_{jk}$ denotes the representative pollutant discharge per unit waste water of water department k of calculation unit j($\mathrm{ton}/m^{3}$), and $p_{jk}$ represents the sewage discharge coefficient of water consumption department k of calculation unit j.

#### 3.1.2. Constraint Setting

(1) Intergenerational equity restriction

The discounted value of economic benefits of water resources allocation in 2050 should not be less than the economic benefits in 2020.

(4) $$\frac{f_{1,2050}}{{\left(1+q\right)}^{30}}\geq f_{1,2020}$$

where $f_{1,2050}$ and $f_{1,2020}$ are the economic benefits in 2050 and 2020, respectively, q is the discount rate.

(2) Water supply restriction

The water supply quantity of each water supply source to each calculation unit shall not exceed the water supply quantity of each water supply source.

(5) $$\sum_{j=1}^{J}\sum_{k=1}^{K}Q_{ijk}\leq WR_{i}$$

where $WR_{i}$ is the available water supply of the first water source.

(3) Water demand constraint

In line with the principle of the saving and effective utilization of water resources, the water supply of each water source to each water department of each calculation unit every month shall not exceed the water demand of each water department of each calculation unit.

(6) $$\sum_{i=1}^{I}Q_{ijk}\leq D_{jk}$$

where $D_{jk}$ is the water demand of the water sector k of the calculation unit j.

(4) Water supply capacity constraints

Each calculation unit has its own water supply capacity for each water source allocation project. The water supply capacity of each water source to each calculation unit’s water consumption department shall not exceed the maximum capacity of water supply of each water source to each calculation unit.

(7) $$\sum_{k=1}^{K}Q_{ijk}\leq U_{\max ij}$$

where $U_{\max ij}$ is the maximum capacity of water supply from the first source to the calculation unit.

(5) Reservoir cap.acity constraints

The storage capacity of each reservoir at the end of the month shall not exceed the maximum storage capacity and shall not be lower than the minimum storage.

(8) $$V_{\min}\leq V_{t}\leq V_{\max}$$

(9) $$V_{t}=V_{t-1}+W_{t-1}-Q_{t-1}$$

where $V_{t}$ is the reservoir capacity at the end of t period; $V_{\min}$ is the minimum storage capacity of the reservoir; $V_{\max}$ is the maximum capacity of the reservoir; $W_{t-1}$ is the inflow of reservoir in t−1 period; $Q_{t-1}$ is the outflow of reservoir in t−1 period.

#### 3.1.3. Global Model

The multi-objective water resources allocation model accounts for the economic benefits generated by water resources allocation and the societal degree of satisfaction for water resources, and takes both as the basis of social sustainability to coordinate social development and the water resources system. It considers the discount value of pollutant emission, ecological water demand and economic benefits to describe an inter-temporal water resources allocation pattern. The multi-objective programming model is obtained by integrating the above objective functions and constraints, as shown in Equation (10).

(10) $$\begin{array}{l} \min f_{1}(Q)={\sum_{j=1}^{J}\sum_{k=1}^{K}\left(\frac{D_{jk}-\sum_{t}^{T}\sum_{i=1}^{I}Q_{ijkt}}{D_{jk}}\right)}^{2}\\ \max f_{2}(Q)=\sum_{t=1}^{T}\sum_{i=1}^{I}\sum_{j=1}^{J}\sum_{k=1}^{K}\left(b_{ijk}Q_{ijkt}-c_{ijk}Q_{ijkt}\right)\\ \min f_{3}(Q)=\sum_{j=1}^{J}\sum_{k=1}^{K}0.1\cdot d_{jk}p_{jk}\sum_{i=1}^{I}Q_{ijk}\\ s.t.\left\{ \begin{array}{l} \frac{f_{1,2050}}{{\left(1+q\right)}^{30}}\geq f_{1,2020}\\ \sum_{j=1}^{J}\sum_{k=1}^{K}Q_{ijk}\leq WR_{i}\\ \sum_{i=1}^{I}Q_{ijk}\leq D_{jk}\\ V_{\min}\leq V_{t}\leq V_{\max} \end{array}\right. \end{array}$$

### 3.2. Modern Solution

In this paper, a method based on setting weights is used to integrate a multi-objective function into a single objective problem [37].

(11) $$\begin{array}{l} \min F(X)=\min\left(\omega_{1}(\frac{f_{1}-f_{1w}}{f_{1o}-f_{1w}})-\omega_{2}(\frac{f_{2}-f_{2w}}{f_{2o}-f_{2w}})+\omega_{3}(\frac{f_{3}-f_{3w}}{f_{3o}-f_{3w}})\right) \end{array}$$

where F is the equality objective function for basin authority; X is the vector for the decision variables; $\omega_{1}$, $\omega_{2}$, and $\omega_{3}$ are weight factors to measure the importance of $f_{1}$, $f_{2}$ and $f_{3}$, and $\omega_{1}+\omega_{2}+\omega_{3}=1$. The objective $f_{o}$ represents the optimal value of f and $f_{w}$ represents the worst value of f.

### 3.3. NSGA-2 Design

The NSGA-2 algorithm for the water resources optimization allocation model has the following steps and, according to the existing literature, NSGA-2 algorithm has good applicability to solve the problem of water resources allocation, and the stability of the model will not be affected by the change of parameters [38,39]:

Step 1: Determine fitness function. Let the negative function of the total objective function be set as the fitness function, namely.

(12) $$Fit(X)=-\left(\omega_{1}(\frac{f_{1}-f_{1w}}{f_{1o}-f_{1w}})-\omega_{2}(\frac{f_{2}-f_{2w}}{f_{2o}-f_{2w}})+\omega_{3}(\frac{f_{3}-f_{3w}}{f_{3o}-f_{3w}})\right)$$

Step 2: Determine the parameter values of NSGA-2. In this paper, the population number is 100, maximum iteration number is 1000, crossover probability is 0.5 and mutation probability is 0.05.

Step 3: Initialize the population and set the iteration number n = 1.

Step 4: Calculate the value of fitness function.

Step 5: Perform crossover and mutation operation for the first-generation population.

Step 6: Integrate the parent and offspring population.

Step 7: Perform non-dominated sorting for the integrated population.

Step 8: Calculate the crowding distance among solutions.

Step 9: Produce a new population from the integrated parent and offspring population by crowding distance.

Step 10: Choose new parent populations by using tournament selection.

Step 11: Check whether the number of iterations reaches the maximum iterations number. If the maximum iterations number is reached, the algorithm is terminated. Otherwise, increase the number of iterations and continue step 5 to step 11.

In this paper, the population number is 100, maximum iteration number is 1000, crossover probability is 0.5 and mutation probability is 0.05 [39,40].

**Figure 3:**
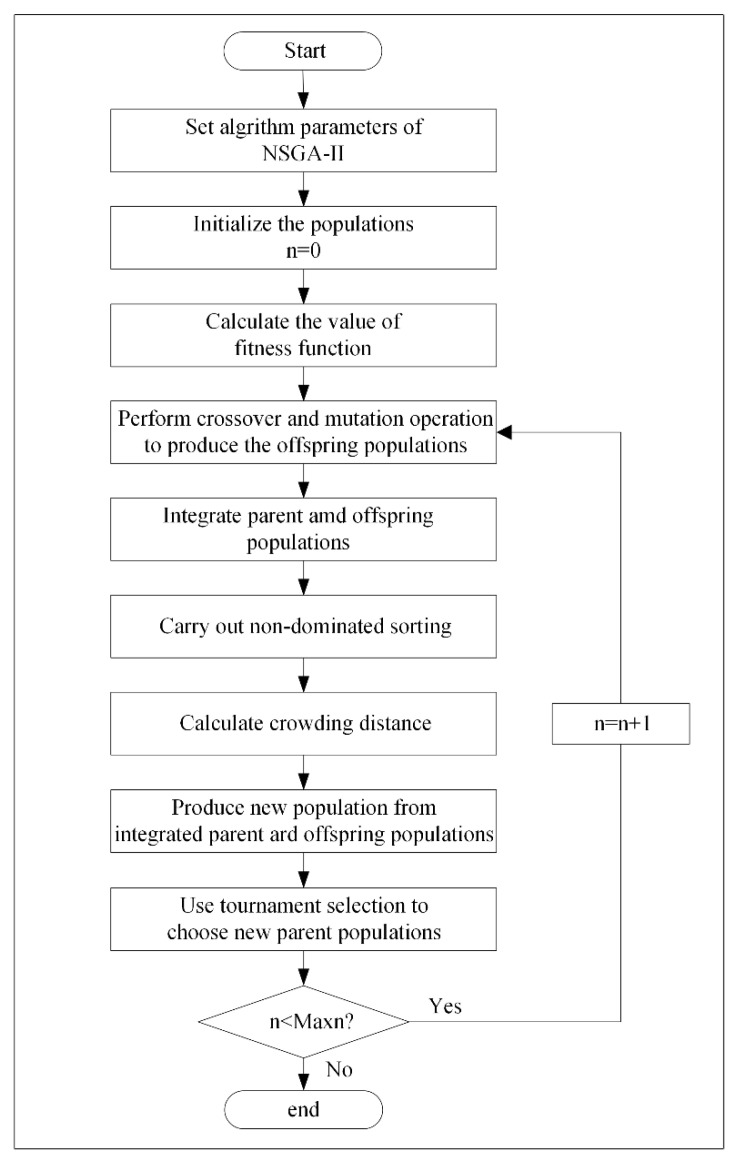
Flow chart of NSGA-2.

The detailed flow chart of the NSGA-2 algorithm to optimize the multi-objective allocation model of water resources with is shown in Figure 3. 

## 4. Result and Recommendation

In this section, the NSGA-2 algorithm is used to calculate the optimal allocation results of water resources in the area of study under different generations and different weights. The practicability of the water resources optimal allocation model considering intergenerational equity is verified and the problem of supply and demand of water in the middle and upper reaches of Huaihe River Basin is solved. Based on the analysis of the calculation results, corresponding suggestions are put forward for water resources management strategy in the middle and upper reaches of the Huaihe River Basin.

### 4.1. Water Demand Forecast of the Middle and Upper Reaches of Huaihe River Basin

According to the actual water use situation of each water department in the Huaihe River Basin, combined with the relevant planning and the statistical yearbook of the Huaihe River Basin, the water demand of domestic, agricultural, production and ecological water departments in 2020 and 2050 is shown in Table 1.

### 4.2. Benefit Analysis

**Figure 4:**
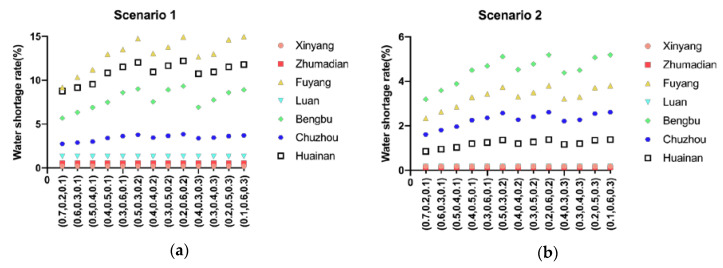
Water shortage rate of different subareas with different sets of objective weights ((**a**) 2020, (**b**) 2050, (0.7 0.2 0.1) means $\omega_{1}=0.7$, $\omega_{2}=0.2$, $\omega_{3}=0.1$).

The water shortage rate represents the social benefits and fairness of water resources allocation. As shown in Figure 4, under different weights, compared with 2020, the water shortage rate in 2050 has decreased to a certain extent, which ensures social stability and equity.

**Figure 5:**
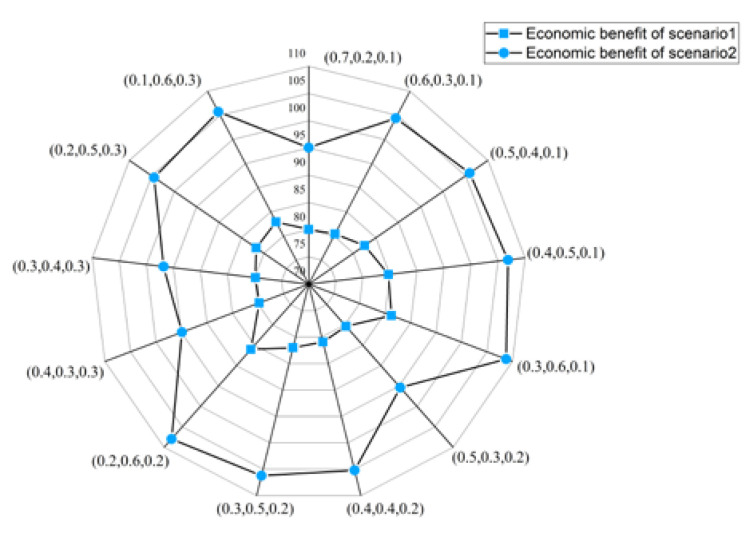
Economic benefits with different sets of objective weights ((0.7, 0.2, 0.1) means $\omega_{1}=0.7$, $\omega_{2}=0.2$, $\omega_{3}=0.1$).

Economic benefits represent the monetary value of the benefits from allocation and embody the efficiency of water resources allocation. Figure 5 shows that although the water resources allocation in 2020 has produced good economic benefits, the economic benefits of water resources allocation in 2050 are far greater than those in 2020. Under various weights, compared with 2020, the economic benefits of 2050 will increase by 20% to 30%, which indicates that the efficiency of water resources allocation in 2050 has been improved.

In addition, environmental benefits and ecological water demand represent the renewability of water resources. In this paper, the model fully meets the ecological water demand and keeps the pollutant emission reasonable, to maintain the renewability of water resources. Meanwhile, the discount value of water resources economic benefits in 2050 is higher than that in 2020, thus showing intergenerational equity.

### 4.3. Total Economic Benefits Analysis

**Figure 6:**
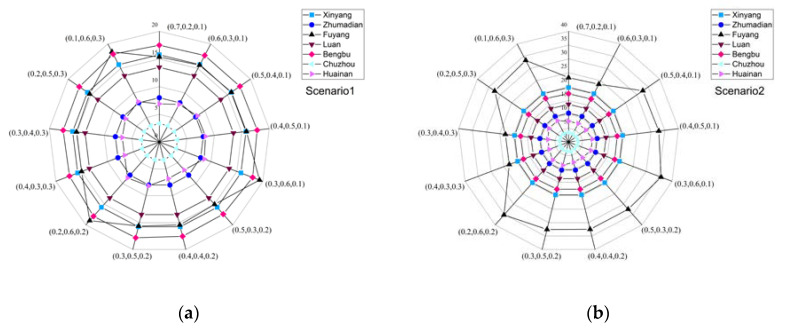
Total economic benefits of different subareas with different sets of objective weights ((**a**) 2020, (**b**) 2050, (0.7 0.2 0.1) means $\omega_{1}=0.7$, $\omega_{2}=0.2$, $\omega_{3}=0.1$).

Figure 6 shows the economic benefits of each subarea in different situations under different weights. Overall, the economic benefits of each subarea are increasing. In 2020, Bengbu has received the maximum economic benefit, followed by Fuyang and Xinyang. In 2050, Fuyang is projected to receive the maximum economic benefit, followed by Xinyang and Bengbu. In 2020 and 2050, except for Fuyang, the economic benefits are stable under different weights of the objective function. In Fuyang, economic benefits will fluctuate under different sets of weights. Fuyang would receive the maximum economic benefit of 19.23(10^10^ Yuan) in 2020, and the maximum economic benefit obtained by Fuyang in 2050 is projected to be 35.98(10^10^ Yuan).

### 4.4. Water Resources Allocation Strategies Analysis of Different Generations

Figure 4 shows the water shortage ratio of the seven cities in the middle and upper reaches of Huaihe River Basin under different generations and with different weights in the objective function. This allows the evaluation of the sensitivity of the seven cities in relation to the objective function. As shown in Figure 4, under the same weight, the water shortage ratio of all the cities of the future generation has decreased to a certain extent compared with that of the current generation. The water shortage ratio of Xinyang, Zhumadian and Lu’an changes little with the changing weight of the objective function, and the water shortage ratio is within 1%. It can be concluded that these three cities are not short of water, so they are not sensitive to the objective function. However, in the contemporary generation, the water shortage rate of Fuyang, Bengbu, Chuzhou and Huainan has changed greatly, as much as 6% in Fuyang. In the future, the water shortage rate of Fuyang, Bengbu and Chuzhou will still change greatly. These cities are all in the middle reaches of the Huaihe River Basin. This shows that the water resources conflicts that area are fierce.

This paper focuses on the analysis of Fuyang, the city with the greatest change of water shortage ratio. In Fuyang, in both the current and future generation, when the weight of economic benefits increases, the water shortage rate will rise, and the water resources allocated to Fuyang will decrease. When the weight of social benefits and environmental benefits increase, the water shortage rate will decrease, and the river basin management department will allocate more water resources to Fuyang. Therefore, the result of water resources allocation depends on the preference of basin management departments for economic and social stability and sustainability.

### 4.5. Results of Considering and Not Considering Intergenerational Equity

**Figure 7:**
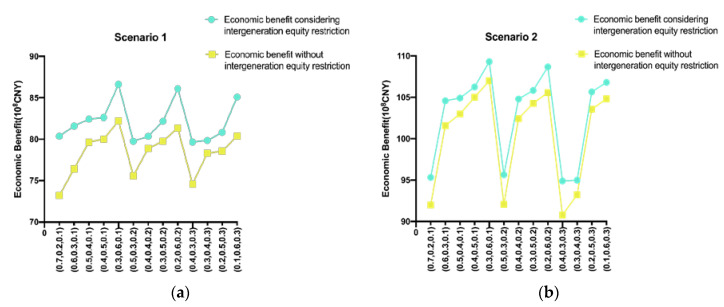
Comparison of economic benefits that do and do not consider intergeneration equity restriction ((**a**) 2020, (**b**) 2050, (0.7 0.2 0.1) means $\omega_{1}=0.7$, $\omega_{2}=0.2$, $\omega_{3}=0.1$).

Figure 7 shows that considering intergenerational equity, the total economic benefit provided by water resources allocation is higher than that provided by the traditional model, when intergenerational equity restrictions are in place. In fact, it is close to the maximum sustainable production, realizing the sustainable development of the study area. The results show that the model guarantees social and economic stability with the minimum water shortage ratio. The discount value of water resources benefits is not lower than that of the previous generation. This means that there will be greater social benefits, a maximum of economic benefits, and the renewability of water resources will be ensured. Therefore, the optimal allocation model of water resources that considers intergenerational equity proposed in this paper is a better solution for river basin management departments, as compared to previous models.

### 4.6. Analysis of Specific Scheme under Balanced Weights ($\omega_{1}=1/3$,$\omega_{2}=1/3$,$\omega_{3}=1/3$)

#### 4.6.1. Specific Water Resources Allocation Scheme under Balanced Weights

**Figure 8:**
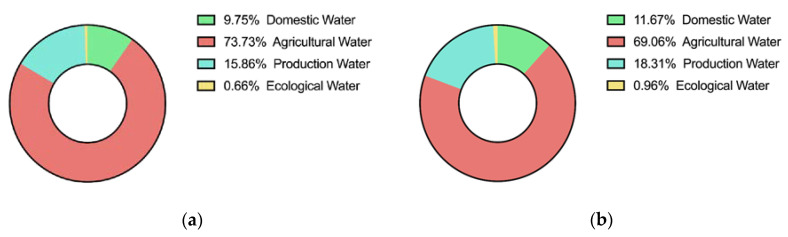
Water consumption proportion of each water department in Huaihe in (**a**) 2020, (**b**) 2050.

**Figure 9:**
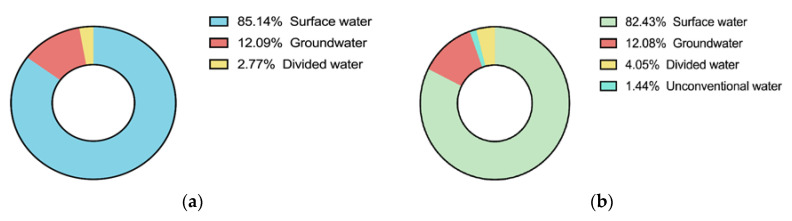
Water supply ratio of each water source in Huaihe in (**a**) 2020, (**b**) 2050.

The NSGA-2 algorithm is used to obtain the optimal allocation scheme of water resources in the middle and upper reaches of Huaihe River Basin in 2020 and 2050. The specific water allocation and proportion of water usage of each department are shown in Table 2 and Figure 8, and the water supply proportion of each water source is shown in Table 3 and Figure 9.

As shown in Figure 8, agricultural water accounts for a large proportion of water usage, reaching 70%. In the future, in order to achieve sustainable development, the middle and upper reaches of the Huaihe River Basin should select better methods of planting, introduce low water consumption and high yield crops, improve the irrigation mode, introduce international advanced irrigation methods, and improve the efficiency of use of water resources.

As shown in Figure 9, the local surface water supplies 80% of the water for seven cities in the middle and upper reaches of the Huaihe River Basin. Compared with 2020, 2050 will reduce the exploitation of groundwater resources, increase external water diversion and unconventional water, protect groundwater resources, and improve water resources sustainability and socially sustainable development capacity.

#### 4.6.2. Social Benefits Analysis

**Figure 10:**
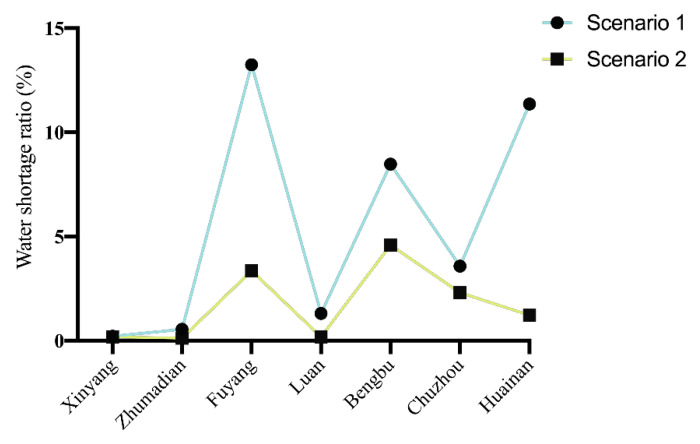
Water shortage rate of 7 cities under balanced weights.

Figure 10 shows the water shortage rate of each subarea under the balanced weight. From 2020 to 2030, the water shortage rate of each subarea is decreasing. The reason for this is that the allocation of water resources plays a coordinating role in the supply and demand of water resources. There will be a corresponding adjustment according to the water source and demand of the research area. In addition, the water transfer scale is expanded due to the Middle Route Project of the South-to-North Water Transfer in China. Therefore, through the optimal management of water resources and some engineering measures, the social benefits of the study area have been improved and the water supply in this area has increased, making the gap between supply and demand smaller.

#### 4.6.3. Economic Benefits Analysis and Intergeneration Equity Analysis

Table 4 shows that the economic benefits in 2050 are projected to be 168.3 (10^10^ Yuan), higher than those in 2020, and the discounted value of economic benefits in 2050 is also projected to be higher than that in 2020. At the same time, the model considers the pollutant emissions and fully meets the ecological water demand to ensure the renewable water resources. Based on these conditions, the model achieves intergenerational equity in the study area.

## 5. Conclusions

This paper illustrates a water resources management strategy, based on intergenerational equity, which can improve the social satisfaction with water resources and achieve higher economic benefits. The proposed water resources allocation model combines temporal and spatial dimensions. It takes the guarantee of water resources renewability as the premise of allocation and the water shortage ratio as the index to measure the degree of satisfaction of society in relation to water resources. This model also uses the discount value of the economic benefits generated by water resources allocation to measure the inter temporal allocation effect of water resources.

The model was verified using seven cities in the middle and upper reaches of the Huaihe River Basin that are taken as the study area. The results show that, under the balanced weight, the water shortage ratio of the study area in 2020 is 5.24%, and 1.58% in 2050, decreasing by 3.66% over time. The economic benefit of water resource allocation significantly increases from 79.46(10^10^ CNY) in 2020 to 168.3(10^10^ CNY) in 2050. In the water use sector, the proportion of agricultural water consumption decreased from 73.73% in 2020 to 69.09% in 2020. Although the proportion of agricultural water use decreased in 2050, it still accounted for a large amount of usage. Ecological water use increased from 0.66% to 0.96%, showing that the river basin management department has increased the importance of water ecology. Regarding water sources, the proportion of surface water supply is 85.14% in 2020, and 82.43% in 2050, a 2.71% decrease. The proportion of groundwater supply decreases slightly from 12.09% to 12.08%. At the same time, the water sources in the area of study will be more abundant in 2050, with unconventional water and water diversion from other basins becoming available in addition to the water sources. This reflects that the river basin management departments will pay more attention to the protection of local water resources and the reasonable allocation of water resources across basins. Analysis of the calculation results show that the model can realize the coordinated, stable and sustainable development of the social system, economic system and water resources system. This can protect the water rights and increase the efficiency, equality, and sustainable development of water resource distribution for future generations.

The optimization allocation model of water resources based on intergenerational equity will continue to be improved. A long series of time scales should be considered, such as the study of the distribution of water resources in ten years, rather than only two typical years of the two generations. Additionally, the current model is mainly aimed at the allocation of water resources quantity. In the long run, water pollution accumulates. After future research, the model will determine the pollutant emissions from the pollutant emission rate of unit water consumption and simulate the pollutant accumulation after creating a series of water environmental protection measures. Therefore, in the future, an intergenerational equity water resources management method considering uncertainty of water resources and integrating both water quantity and quality is to be developed.

## Figures and Tables

**Table 1:** Forecast of water demand in 2020 and 2050 (10^8^ m^3^).

**Scenario 1**	**Domestic Water Demand**	**Agricultural Water Demand**	**Production Water Demand**	**Ecological Water Demand**	**Total**
Xinyang	2.30	31.05	2.69	0.11	36.16
Zhumadian	2.69	8.02	3.31	0.11	14.13
Fuyang	3.67	23.04	5.14	0.24	32.10
Luan	1.99	21.55	4.23	0.14	27.90
Bengbu	1.99	11.34	5.64	0.19	19.15
Chuzhou	0.61	5.73	0.83	0.04	7.21
Huainan	1.08	6.71	1.55	0.10	9.44
**Scenario 2**	**Domestic Water Demand**	**Agricultural Water Demand**	**Production Water Demand**	**Ecological Water Demand**	**Total**
Xinyang	3.28	31.23	4.02	0.24	38.77
Zhumadian	3.15	8.35	3.38	0.16	15.04
Fuyang	3.83	20.72	5.96	0.36	30.88
Luan	2.14	17.87	4.23	0.17	24.40
Bengbu	2.17	8.37	5.79	0.23	16.56
Chuzhou	0.70	5.62	0.90	0.05	7.26
Huainan	1.20	5.03	1.66	0.12	8.01

**Table 2:** Specific allocation scheme in Huaihe River Basin in 2020 and 2050 (10^8^ m^3^).

**Scenario 1**	**Domestic Water**	**Agricultural Water**	**Production Water**	**Ecological Water**	**Total**
Xinyang	2.30	30.99	2.69	0.11	36.08
Zhumadian	2.67	7.98	3.29	0.11	14.05
Fuyang	3.18	19.97	4.46	0.24	27.85
Luan	1.96	21.22	4.16	0.14	27.48
Bengbu	1.82	10.37	5.16	0.19	17.53
Chuzhou	0.59	5.54	0.80	0.04	6.97
Huainan	0.97	6.01	1.39	0.10	8.46
**Scenario 2**	**Domestic Water**	**Agricultural Water**	**Production Water**	**Ecological Water**	**Total**
Xinyang	3.28	31.18	4.01	0.24	38.70
Zhumadian	3.15	8.33	3.37	0.16	15.01
Fuyang	3.70	20.02	5.76	0.36	29.84
Luan	2.13	17.84	4.22	0.17	24.36
Bengbu	2.07	7.98	5.52	0.23	15.80
Chuzhou	0.68	5.49	0.88	0.05	7.10
Huainan	1.18	4.95	1.64	0.12	7.89

**Table 3:** Water supply of each water source in Huaihe River Basin in 2020 and 2050 (10^8^ m^3^).

**Scenario 1**	**Surface Water**	**Ground Water**	**Divided Water**	**Unconventional Water**	**Total**
Xinyang	34.9	1.18	0	0	36.08
Zhumadian	11.32	2.73	0	0	14.05
Fuyang	16.34	7.69	0	3.83	27.85
Luan	27.18	0.29	0	0	27.48
Bengbu	14.28	3.25	0	0	17.53
Chuzhou	6.62	0.35	0	0	6.97
Huainan	7.21	1.25	0	0	8.46
**Scenario 2**	**Surface Water**	**Ground Water**	**Divided Water**	**Unconventional Water**	**Total**
Xinyang	37.45	1.18	0	0	38.7
Zhumadian	12.28	2.73	0	0	15.01
Fuyang	16.66	8.09	0	5.1	29.84
Luan	24.07	0.29	0	0	24.36
Bengbu	12.64	3.15	0	0	15.8
Chuzhou	5.96	0.15	1	0	7.1
Huainan	5.22	1.15	1	0.52	7.89

**Table 4:** Economic benefit and discount value (10^10^ CNY).

	Economic Benefits	Discount Value of Economic Benefits
Scenario 1	79.46	79.46
Scenario 2	168.3	80.23
